# Perfidious synaptic transmission in the guinea-pig auditory brainstem

**DOI:** 10.1371/journal.pone.0203712

**Published:** 2018-10-04

**Authors:** Arkadiusz Stasiak, Mark Sayles, Ian M. Winter

**Affiliations:** Centre for the Neural Basis of Hearing, The Physiological Laboratory, Downing Street, Cambridge, United Kingdom; Universidad de Salamanca, SPAIN

## Abstract

The presence of ‘giant’ synapses in the auditory brainstem is thought to be a specialization designed to encode temporal information to support perception of pitch, frequency, and sound-source localisation. These ‘giant’ synapses have been found in the ventral cochlear nucleus, the medial nucleus of the trapezoid body and the ventral nucleus of the lateral lemniscus. An interpretation of these synapses as simple relays has, however, been challenged by the observation in the gerbil that the action potential frequently fails in the ventral cochlear nucleus. Given the prominence of these synapses it is important to establish whether this phenomenon is unique to the gerbil or can be observed in other species. Here we examine the responses of units, thought to be the output of neurons in receipt of ‘giant’ synaptic endings, in the ventral cochlear nucleus and the medial nucleus of the trapezoid body in the guinea pig. We found that failure of the action-potential component, recorded from cells in the ventral cochlear nucleus, occurred in ~60% of spike waveforms when recording spontaneous activity. In the medial nucleus of the trapezoid body, we did not find evidence for action-potential failure. In the ventral cochlear nucleus action-potential failures transform the receptive field between input and output of bushy cells. Additionally, the action-potential failures result in “non-primary-like” temporal-adaptation patterns. This is important for computational models of the auditory system, which commonly assume the responses of ventral cochlear nucleus bushy cells are very similar to their “primary like” auditory-nerve-fibre inputs.

## Introduction

The auditory system is anatomically and physiologically specialized for rapid and precise temporal coding [1–3]. Temporal information is thought to be critical for neural representations of sound-source location, the pitch of musical sounds, and spectral features of complex sounds, such as speech [4–11]. Synchronization of auditory-nerve-fibre (ANF) action potentials to the oscillations of the band-limited cochlear output is the neural basis for peripheral encoding of temporal acoustic features [12–14]. The temporal information in ANF firing patterns is exploited by specialized auditory brainstem circuits.

The presence of “giant” synapses in auditory brainstem circuits is typically thought to represent a morphological specialization critical for highly reliable synaptic transmission, and to preserve or even enhance the temporal information first encoded in ANFs. These specialised synapses exist at several key brainstem processing locations in the ascending auditory pathway: the ventral cochlear nucleus (VCN), the medial nucleus of the trapezoid body (MNTB), and the ventral nucleus of the lateral lemniscus (VNLL). These neural circuits are involved in processing the high-fidelity temporal information arriving as stimulus-structure synchronized action potentials in ANFs. In the VCN, ANFs form giant “endbulb of Held” synapses onto spherical bushy cells, and modified endbulb synapses onto globular bushy cells [15]. These two populations of VCN principal neurons project via the trapezoid body to anatomically distinct cells in the superior olivary complex (SOC). Neurons in the medial and lateral superior olives (MSO and LSO, respectively) receive synaptic input from the VCN and MNTB, synchronised to the ongoing structure of sounds at the two ears. To encode sound-source location, principal cells in the MSO and LSO perform across-ear coincidence-detection and anti-coincidence-detection operations on these inputs.

Physiological evidence is, however, emerging from several mammalian species (mouse, gerbil) that auditory brainstem giant synapses are not simply reliable “one-to-one relays”. Because these axo-somatic synapses are so large, extra-cellularly recorded potentials are complex with three distinct components from the separate elements of the synapse. The components are thought to correspond to the pre-synaptic potential (PP), the excitatory post-synaptic potential (EPSP), and the evoked action potential (AP), all recorded extra-cellularly [16–22]. Traditionally, these giant synapses have been thought to allow secure transmission. Quite remarkable is the observation that these giant synapses frequently fail. That is, extra-cellularly recorded potentials have revealed that often only the PP and EPSP components occur, with no AP. This failed synaptic transmission likely has important functional implications for auditory brainstem neural circuits.

Here, we report the properties of extra-cellularly recorded potentials in the VCN and MNTB of the anaesthetised guinea pig. The guinea-pig is an important animal model of peripheral and central auditory processing because of its ability to hear low-frequency sounds in the range of human speech and music, and its ability to encode temporal information over a similar range of frequencies as that estimated in humans [23]. Using a quantitative procedure to determine spike-waveform shape we demonstrate that guinea-pig VCN units with a PP in their extra-cellular spike waveform are characterised by a high probability of AP failure. This failure is manifest in the non-primary-like receptive field of these units in response to acoustic stimulation, and also in the absence of a primary-like temporal adaptation pattern. In contrast, PP units in the MNTB are characterised by reliable synaptic transmission, with zero AP failures. Our data provide further evidence to support recent findings from other mammalian species. In particular, we find a high probability of synaptic transmission failure at endbulb of Held synapses in the VCN, similar to the gerbil [16, 24] and cat [19]. Transmission does not fail in our population of neurons in the guinea-pig MNTB. This finding is similar to data in the gerbil [25] and cat [21], but differs substantially from the mouse [22]. The perfidiousness of transmission at giant synapses should be included in future models of auditory brainstem processing.

## Methods

### Ethical approval

All animal experiments were approved by the University of Cambridge ethical review committee, as regulated by UK legislation, set out in the Animals (Scientific Procedures) Act. All personnel held personal licenses, issued by the UK Home Office. The experiments were carried out under the auspices of two different UK Home Office project licenses to IMW (8002245, 7007702). All procedures complied with accepted ethical best practice. Animals were obtained from our in-house breeding colony, maintained in accordance with relevant UK legislation. Animals were fed *ad libitum*.

## Animal preparation

Experiments were performed on 89 pigmented guinea pigs (*Cavia porcellus*), weighing between 320 and 850 g. In 86 animals, the VCN was targeted. In the remaining 3 animals, data were obtained in the MNTB. All surgical and recording procedures were performed inside an electrically and acoustically-shielded chamber (Industrial Acoustics Company, Winchester, UK). Incisions were pre-infiltrated with 2% lidocaine. Animals were anaesthetized with urethane (1.0 g/kg, *ip*). Hypnorm (*fentanyl citrate*, 0.315 mg/ml; *fluanisone*, 10 mg/ml; Janssen, High Wycombe, UK) was administered as supplementary analgesia (1 ml/kg, *im*). Anesthesia and analgesia were maintained at a depth sufficient to abolish the pedal withdrawal reflex (front paw). Additional doses of Hypnorm (1 ml/kg, *im*) or Urethane (0.5 g/kg, *ip*) were administered on indication. Core temperature was monitored with a rectal probe and maintained at 38°C using a thermostatically-controlled heating blanket (Harvard Apparatus, MA). The trachea was cannulated and on signs of suppressed respiration, the animal was ventilated with a pump (Bioscience, UK). The animal was placed in a stereotaxic frame, which had ear bars coupled to hollow speculae designed for the guinea-pig ear. A mid-sagittal scalp incision was made and the periosteum and the muscles attached to the temporal and occipital bones were removed. A silver-coated wire contacted the cochlea, near the round window, for monitoring compound action potentials (CAP). The CAP threshold was determined at selected frequencies at the start of the experiment and thereafter upon indication. If CAP thresholds deteriorated by more than 10 dB and were non-recoverable (e.g., by removing fluid from the bulla or by artificially ventilating the animal), the experiment was terminated (using an overdose of sodium pentobarbital; Euthetal, May & Baker, Dagenham, UK). A craniotomy was fashioned, exposing the left cerebellum. The overlying dura was removed and the left cochlear nucleus was exposed via an aspiration cerebellotomy. The cerebellotomy was filled with 1.5% agar-in-saline (Sigma-Aldrich, UK) to prevent desiccation and to aid recording stability.

### Neural recordings

Single-unit responses were recorded extracellularly with glass-coated tungsten microelectrodes [26]. Electrodes were advanced in the sagittal plane by a hydraulic microdrive (650 W; David Kopf Instruments, Tujunga, CA) at an angle of 45°. Single units were isolated using broadband noise as a search stimulus. All stimuli were digitally synthesized in real-time with a PC equipped with a DIGI 9636 PCI card that was connected optically to an AD/DA converter (ADI-8 DS, RME audio products, Germany). The AD/DA converter was used for digital-to-analog conversion of the stimuli as well as for analog-to-digital conversion of the amplified (x 1000) and filtered (0.3–10 kHz) neural activity. The sample rate was 96 kHz. The AD/DA converter was driven using ASIO (Audio Streaming Input Output) and SDK (Software Developer Kit) from Steinberg [27].

After digital-to-analog conversion, the stimuli were equalized (phonic graphic equalizer, model EQ 3600; Apple Sound) to compensate for the speaker and coupler frequency response and fed into a power amplifier (Rotel RB971) and a programmable end attenuator (0–75 dB in 5 dB steps, custom build) before being presented over a speaker (Radio Shack 40–1377 tweeter assembled by Mike Ravicz, MIT, Cambridge, MA) mounted in the coupler designed for the ear of a guinea pig. The stimuli were monitored acoustically using a condenser microphone (Brüel & Kjær 4134, Denmark) attached to a calibrated 1-mm diameter probe tube that was inserted into the speculum close to the eardrum. Neural spikes were discriminated in software, stored as spike times on a PC and analyzed off-line using custom-written MATLAB programs (The MathWorks, Inc., Natick, MA).

Upon isolation of a unit, its BF and excitatory threshold were determined using audio-visual criteria. Spontaneous activity was measured over a ten-second period. Single units were classified based on their peri-stimulus time histograms (PSTH), the first-order interspike-interval distribution and the coefficient of variation (CV) of the discharge regularity. The CV was calculated by averaging the ratios of the mean ISI and its standard deviation between 12 and 20 ms after onset. PSTHs were generated from spike-times collected in response to 250 sweeps of a 50-ms tone at the unit’s BF at 20- and 50-dB above threshold. Tones were presented with randomized starting phase, and repeated with a 250-ms period. PSTHs were classified as primary-like (PL), primary-like with a notch (PN), chopper-sustained (CS), chopper-transient (CT), and onset-chopper (OC). All units were classified based on the PSTH shape produced by triggering on the AP component of the spike waveform. For units where the AP component failed a large number of times, this has led to a further classification of unusual (UN).

### Analyzing spike waveform shape

To analyse spike-waveform shape, we sampled single-unit spontaneous activity extra-cellularly for 62.5 seconds. Spike waveforms were also sampled in response to 20dB and 50dB supra-threshold BF tone-bursts and, in some cases, when collecting data for the frequency tuning response area.

The main assumption we make when reporting the data is that the recordings were made from a single unit. Ultimately this is impossible to prove, however, we can ensure that recordings which are characterised by spikes occurring within the refractory period are rejected. To this end we adopted the spike sorting paradigm used by McLaughlin *et al* [21] for analysing spike waveform shape in the MNTB of the cat. It should be noted that other spike sorting methods (e.g. [25]) have been used with similar results. Supporting observations that argue for the presence of just one unit rather than two include the fact that the spikes were always lost together. In addition, both 2- and 3-component spike waveform patterns discharged to the same input frequencies and they shared a common best-frequency.

## Results

We recorded spike waveforms from 235 units in the ventral cochlear nucleus (VCN) and 20 units from the Medial Nucleus of the Trapezoid Body (MNTB). For most units, we recorded spontaneous activity and responses to pure tone bursts. These data were collected from 89 animals used primarily for other studies of auditory signal processing (e.g., [6–8, 28]). For comparative purposes, we examined the spike waveform shape of units with a PP, and some without a PP.

### Basic waveform properties

**Fig 1:**
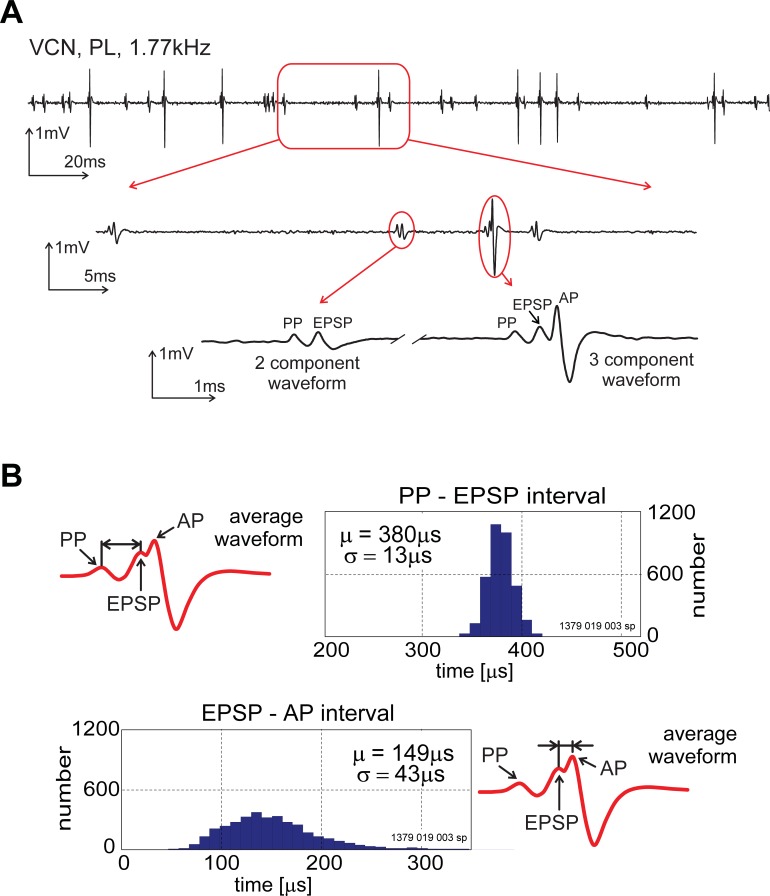
Complex waveforms recorded from spontaneous activity from a single unit in the ventral cochlear nucleus. ***A*,** The plot shows three different time scales of spontaneous activity from a single unit. The longest timescale (top trace) shows what appear to be two discrete spike waveforms. This is confirmed by examining the same spike trace in two shorter time windows. The largest spikes show the traditional 3 component waveform whereas the smaller spikes have the PP and EPSP components but the AP component is absent. ***B*,** The upper plot shows the distribution of intervals between the PP and presumed EPSP components [PP-EPSP interval] for a single unit. The lower plot shows the distribution of intervals, for the same single unit, between the EPSP and AP components [EPSP-AP interval].

For units with spontaneous activity, we searched for the presence of a PP by averaging spike waveforms from the spontaneous activity. For units with no (or very low) spontaneous activity, we used responses to low-intensity best-frequency tone bursts. The mean spontaneous discharge rate of primary-like units with a PP was 46 spikes/s (n = 71) and the unit BFs ranged from 0.35–19.74 kHz. An example of spontaneous activity recorded from a primary-like unit is shown in Fig **1A** for three different time scales. The upper trace shows the longest time period and it is clear that there are two distinct spike amplitudes. The lowest trace (shortest time period) shows that the small amplitude spike contains two peaks while the large spike contains three peaks; the first peak, corresponding to the pre-synaptic, PP, component and two presumed post-synaptic components, EPSP and AP [20]. The main difference between the 2 and 3-component waveforms was the absence of the AP component. We assume that the two waveform shapes are recorded from the same neuron. For the three-component waveform we show in Fig **1B** the distribution of intervals between the PP and EPSP component and the EPSP and the AP component. The time between the peak of the presynaptic activity and the peak of the somatic action potential is approximately 0.5 ms. This delay consists of the 380μs mean-time difference between the pre-synaptic component and the EPSP and the shorter mean-delay of 149 μs between the EPSP and the somatic action potential.

### Classification of single units on the action-potential component of the spike waveform

**Fig 2:**
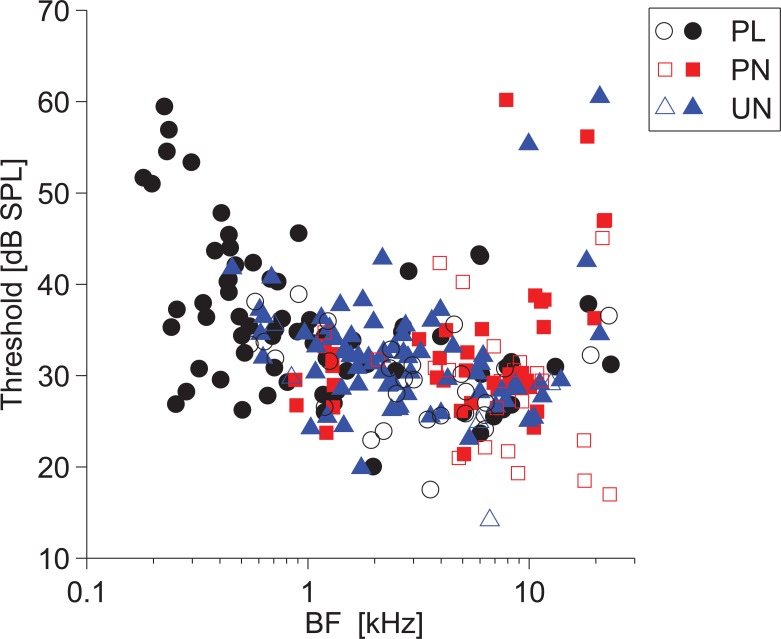
Unit thresholds as a function of BF. PL, primary-like; PN, primary-like with a notch; UN, unusual. Solid symbols indicate unit had an identifiable pre-potential in its averaged spike waveform. Open symbols are non-PP units.

All single units from the VCN and MNTB have been classified by the shape of their post-stimulus time histogram (PSTH) in response to 20-dB supra-threshold BF tone bursts. The PSTHs were constructed from the spike times based upon discrimination of the action-potential-component of the spike waveform. The thresholds at BF for the population of units recorded in this study are shown in Fig **2**. A large number of units (~30%) are classified as unusual (UN); they are intermingled amongst the other unit types. Unusual units are classified as those having a steady-state discharge rate below 70 spikes per second. As in previous studies there is a clear dependency of threshold on BF and as such our population of units is a typical sample from the VCN of the anaesthetised guinea pig. We have identified 184 units with a pre-potential (filled symbols) and they can occur across the different unit groups and across unit BF.

**Fig 3:**
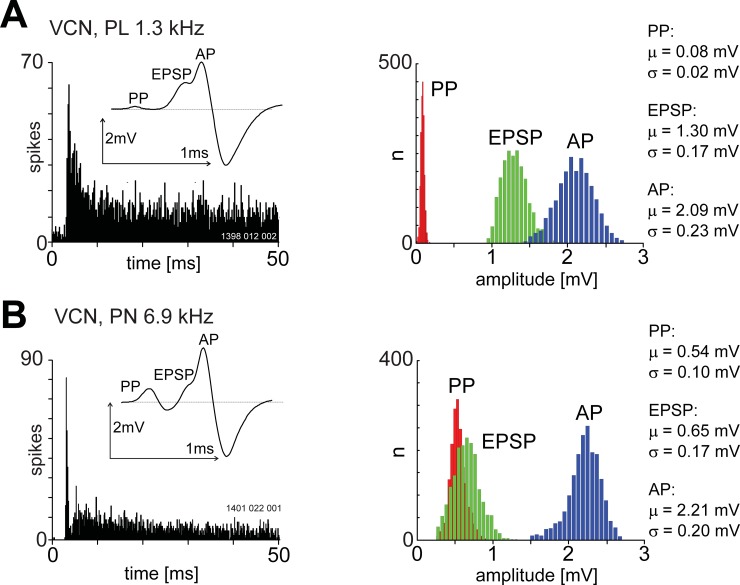
The amplitude distributions for the PP, EPSP and AP components for a primary-like (A) and primary-like with a notch (B) unit recorded from the ventral cochlear nucleus. The unit BF is shown in the upper left of each PSTH. Bin-width was 0.2 ms and sound level was 20 dB above the BF threshold. The stimulus onset was at zero milliseconds and stimulus duration was 50 ms. Inset in the PSTH shows the 3-component averaged spike waveform. The primary-like unit is characterised by a low amplitude for the PP but a much higher mean amplitude for the EPSP. In contrast the PP and EPSP components are very similar for the primary-like with a notch unit. The amplitudes of the AP component are similar for both units.

The amplitude distribution for the three waveform components is shown in Fig **3** for exemplar PL and PN units. All the distributions are approximately symmetrical with the main difference being the larger PP component for the PN compared to the PL unit. Importantly, we did not find multi-modal amplitude distributions for the pre-potential, as might be expected for a quantal process if a bushy cell receives multiple endbulb synapses.

**Fig 4:**
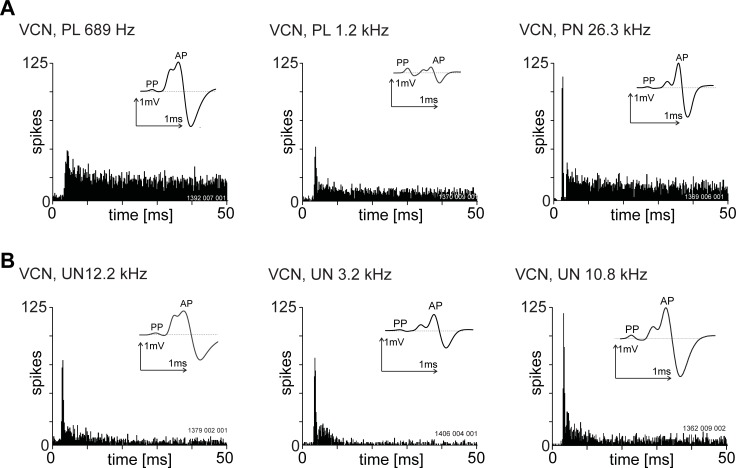
Variety of post stimulus time histogram shapes in response to 20 dB, suprathreshold, BF tone bursts from single units recorded from the ventral cochlear nucleus and with a pre-potential. All histograms were constructed from spike times recorded from the AP component. The unit BF is shown in the upper left of each PSTH. Bin-width was 0.2 ms. Inset in each PSTH is the averaged spike waveform shape. The stimulus onset was at zero milliseconds and stimulus duration was 50 ms. The top row shows three primary-like and primary-like-with-notch units and the bottom row shows three unusual units. Unusual units were arbitrarily identified as those with a steady-state discharge rate (30–40 ms post-stimulus onset time) less than 70 sp/s. The unit BF is indicated above each histogram.

In extra-cellular recordings we conventionally record the presence of an action potential by noting the time the AP-component crosses a spike-discrimination threshold. For most units in the VCN and MNTB this is uncontroversial, however, for the group of units with a PP the AP-component can fail a large percentage of the time resulting in some unusual temporal adaptation patterns. For units with a PP examples of the different PSTH shapes and their corresponding waveforms are shown in Fig **4**. The upper row illustrates units we have classified as primary-like and primary-like-with-notch and the lower row illustrates units which we identify as unusual. For unusual units there is very little discharge approximately 10 ms after stimulus onset. This is a typical pattern seen for units characterised by frequent failures of the AP-component.

**Fig 5:**
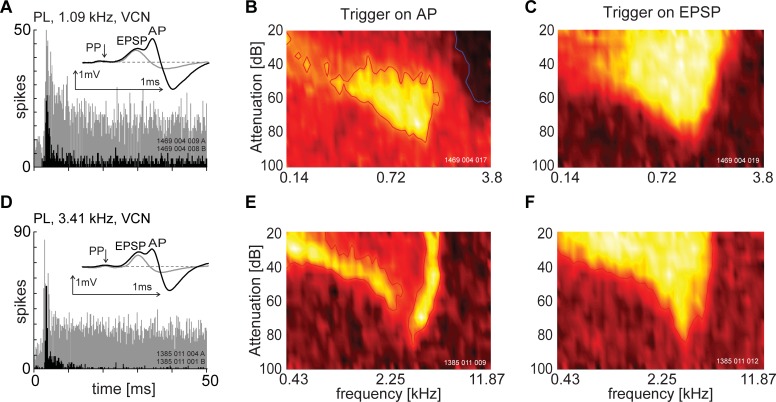
Change in receptive field and temporal adaptation pattern by altering spike discrimination level from the action potential to the excitatory post-synaptic potential. Upper post-stimulus time histogram is primary-like when triggered from the EPSP (grey). The receptive field was type-I (C). When triggered from the AP there are large areas of few spikes in the receptive field (B) and the PSTH (black) is now dominated by the large onset response. The lower PSTH shape is also primary-like when triggered on the EPSP (grey) and the receptive field is type-I. When triggering off the EPSP the PSTH (black) is dominated by an onset component and the receptive field is dominated by a large area in the middle which is reduced in firing rate. The stimulus onset was at zero milliseconds and stimulus duration was 50 ms. The receptive fields are normalised using a pseudo-colour plot where the lighter areas indicate greater activity and the darker areas less activity. The insets in Figs A and D show the average spike waveforms when triggered off the EPSP (grey) and the AP (black).

The shape of a unit’s receptive field can change depending on whether one is triggering on the EPSP (input) or AP (output) component. Fig **5** shows the PSTH and receptive field of two unusual units based on triggering from the AP (black histograms). When triggering on the EPSP component, both PSTHs are conventional in shape, and would be classified as primary-like (grey histogram). Therefore, the classification would be different depending on which potential was discriminated by the spike trigger. The average spike waveform is shown inset with the PSTHs. There is a clear 2-component waveform obtained by averaging spikes that were discriminated on the positive going slope of the EPSP (Fig **5A and 5D** **inset, grey lines**). If the triggering was switched to the action potential, the PSTH was unusual in shape, although the waveform (inset of the PSTH) was a classic 3-component shape (Fig **5A and 5D** **inset, black lines**). The receptive fields were monotonic with sound level, similar to those recorded from ANFs and would be classified as Type I in the VCN when produced by triggering on the EPSP component (Fig 5C and 5F). When triggering was switched to the AP component the receptive field is non-monotonic with sound level, and could be more accurately described as type IV in the DCN receptive field classification scheme [29]. For the unit shown in Fig **5A**, the AP-component is absent over a wide frequency range and is almost totally absent at frequencies above 3 kHz. In contrast the receptive field in Fig **5E** shows a large reduction in discharge rate centred on the unit BF. Such a response was originally described by Winter and Palmer [30] and termed “centre-band suppression”. It is clear from these results that the apparent response of the neuron obtained from extra-cellular recording in the AVCN depends very clearly on whether spike events are discriminated on the EPSP or somatic action potential. By altering the trigger, the data obtained could either reflect the input to, or the output from, a single neuron.

### The complex waveform as a measure for post-synaptic excitability

**Fig 6:**
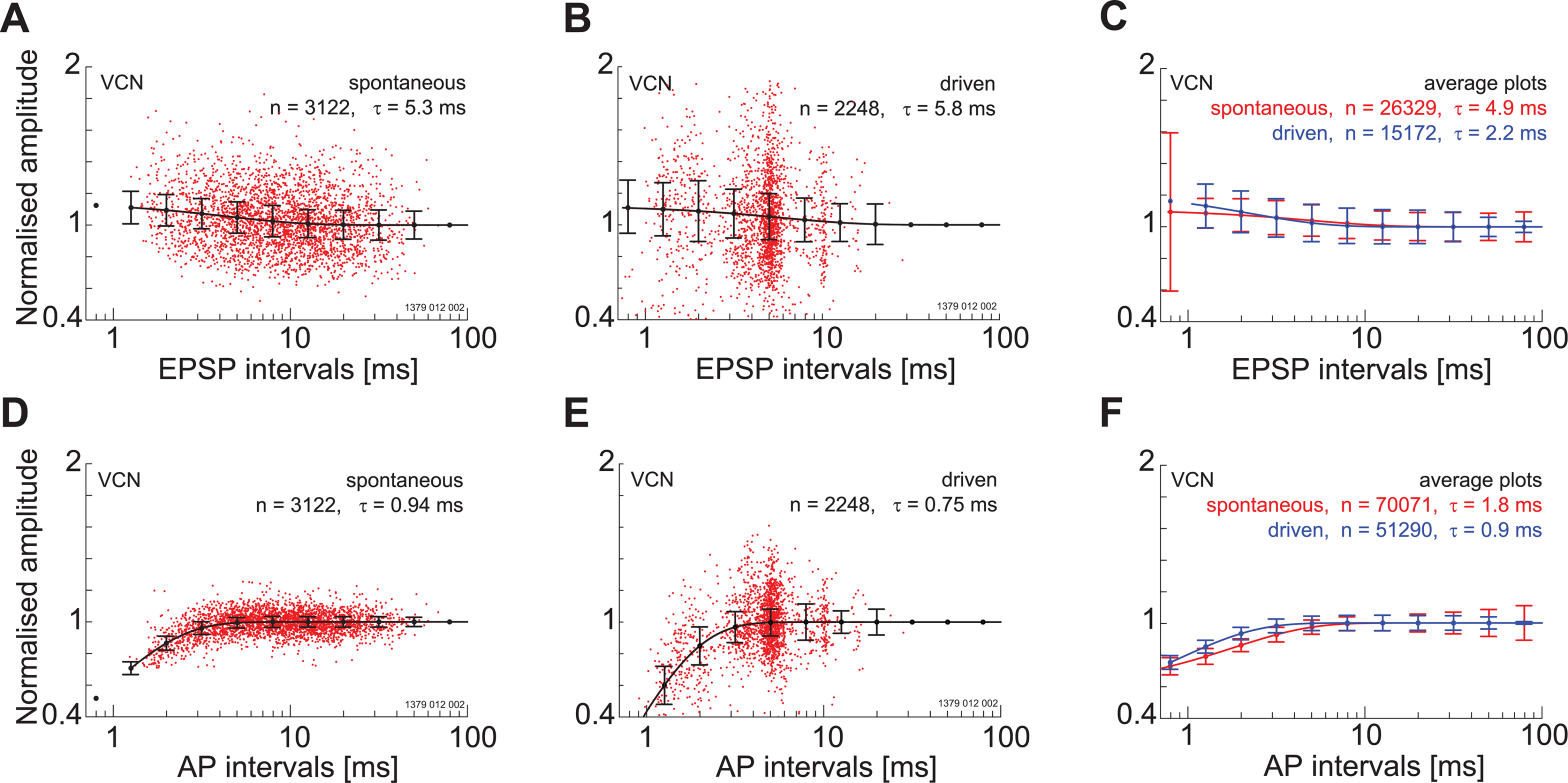
Amplitude of post-synaptic potentials depends on recent history in the ventral cochlear nucleus. Relationship between both EPSP and AP component amplitude as a function of component interval for spontaneous activity (A, D) and driven activity (B,E) for a PL unit; BF = 0.2 kHz. Note that there is no evidence for depression of the EPSP component whereas the amplitude of the AP component decreases at short inter-component intervals. C and F show a population response for the same component intervals. There is a facilitation of the EPSP component amplitude and a depression of the AP component amplitude at the shortest intervals. Vertical lines are standard deviations.

In a recent study in the mouse MNTB it was shown that the amplitude of the extracellular EPSP was a good predictor of the synaptic transmitter release [22]. Unexpectedly, it was also shown that the amplitude of the EPSP was not dependent on spike history; i.e., inter-spike interval. This was despite that fact that *in-vitro* the young adult calyx of Held shows strong synaptic depression. To determine if synaptic depression could influence the size of the EPSP in our data we plot the amplitude of the EPSP component as a function of the EPSP component intervals for a single neuron and a population of neurons located in the VCN. For this single unit, there is no indication that the amplitude of the EPSP-component decreases with decreasing inter-event intervals. In fact, there appears to be a small facilitation at the shortest intervals (Fig **6A and 6B**). This facilitation is also seen when the data are averaged over multiple neurons (Fig **6C**). The amplitude of the AP-component does show signs of suppression at the shortest inter-event intervals (Fig **6D and 6E**). The time course of recovery from this suppression was fitted with a single exponential with a time constant of around 1 ms.

**Fig 7:**
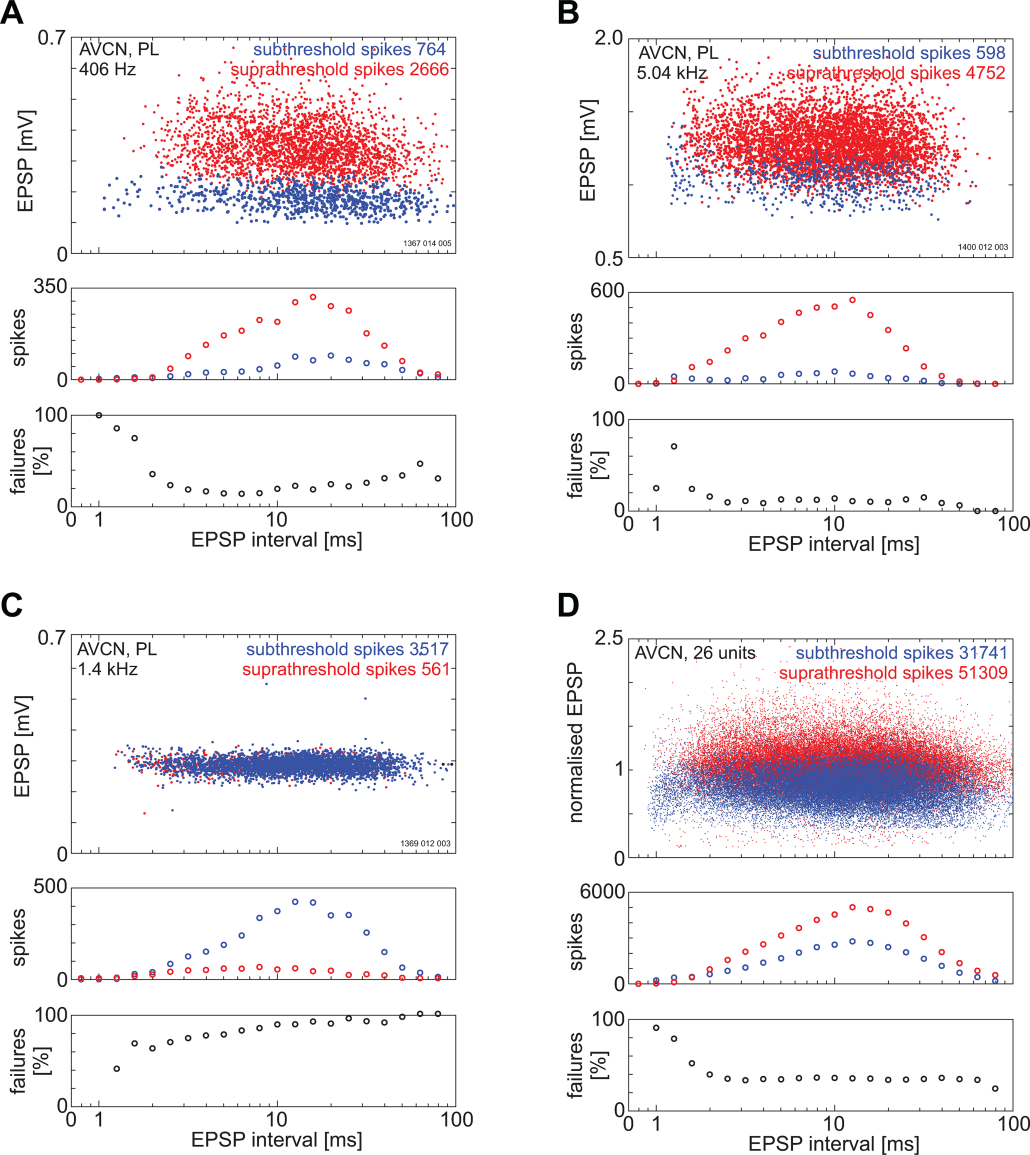
The relationship between EPSP, action potential and EPSP interval in the ventral cochlear nucleus. Note that EPSP amplitudes were normalised by dividing by the mean amplitude of the three longest binned intervals. Three single units have been selected to illustrate the range of responses (A-C) and a population of units averaged in (D). Red dots indicate the EPSPs that were accompanied by an AP i.e. were suprathreshold; Blue dots represent EPSPs that failed to elicit an AP i.e. subthreshold. In A there is a slight decline in the percentage of failures with increasing EPSP interval. In B there are relative few failures although they are fairly evenly spaced as a function of EPSP interval. In C the failures outnumber the successes and increase with increasing EPSP interval. A summary for 26 units is shown in panel D. The number of failures is greatest at the smallest EPSP intervals and then plateaus around an EPSP interval around 3 ms.

There is a clear relationship between the amplitude of the EPSP-component and failings of the AP-component. This is shown in Fig **7** for three single units and a population of 26 AVCN units. In these plots the amplitude of EPSPs that failed to produce a somatic action potential (AP-component) are plotted as blue dots while EPSP amplitudes that were accompanied by a somatic action potential are plotted as red dots. On average the proportion of spike failures is roughly constant as a function of EPSP interval, however, for some units (e.g. Fig **7C**) there is a steady rise in failings with increasing EPSP interval. It is clear from these results that a decrease in EPSP-amplitude leads to an increase in spike failures.

**Fig 8:**
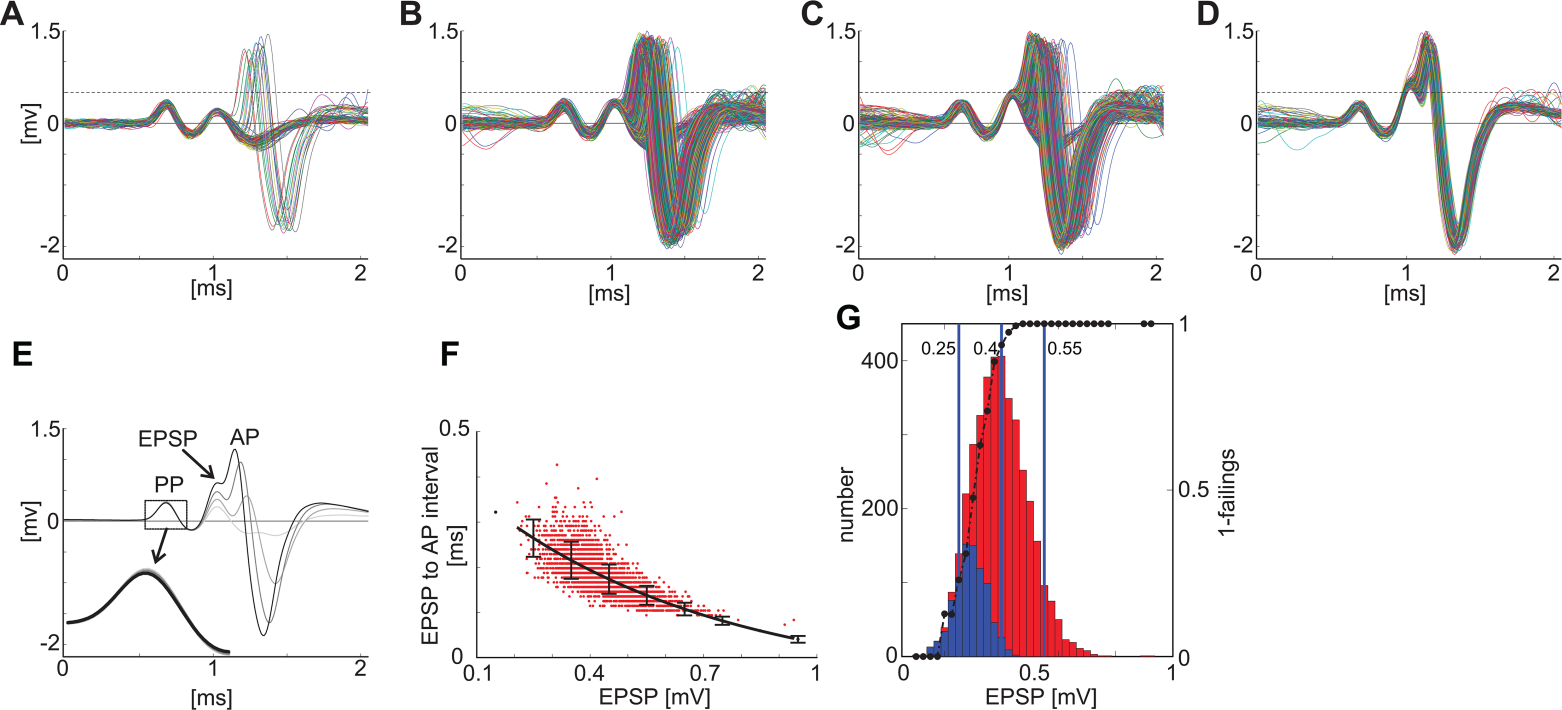
The smaller the EPSP component the more likely the AP component will fail. The first row (A-D) shows the individual spike waveforms for 4 different amplitude ranges for the EPSP component for a single unit (Unusual unit; BF = 1.15kHz). Dotted lines are for visual guide only. For the lowest amplitude (A) the AP-component occurs only occasionally whereas for the highest amplitude (D) the AP-component never fails. This is summarised in the figure in the second row (E). The EPSP-AP interval decreases with increasing EPSP-amplitude (F). G shows the distribution of EPSP-amplitudes when accompanied by an AP-component (red bars) or not followed by an AP component (i.e. failures–blue bar). The dotted black line is the ratio of failures. At high EPSP- amplitudes the unit doesn’t fail.

This is further illustrated in Fig **8** for a single unit which shows that as the amplitude of the EPSP-component increases the probability of the AP-component failing decreases. Fig **8A** shows the spike waveforms for a single neuron when the amplitude of the EPSP component was less than 0.25 mV. For some spikes there is a clear AP-component but for many others the AP-component is absent. Fig **8B–8D** show the proportion of AP-component failures decreases as the amplitude of the EPSP component increases. In Fig **8D** where the amplitude of the EPSP-component is greater than 0.55 mV there are no spike failures. These results are summarised in Fig **8E** which shows the average spike waveform for 4 different ranges of EPSP-component amplitude. Also in this figure is an inset showing that there is little change in the size of the pre-potential despite the increase in EPSP amplitude. The EPSP-AP interval also decreases with an increase in EPSP component amplitude (Fig **8F**). The relationship between EPSP-component amplitude and AP-component failings is summarised in Fig **8G**. The amplitudes of the EPSP-component that were not followed by an AP-component spike are plotted as blue bars while red bars show the amplitudes that were followed by an AP-component spike. The proportion of spike waveforms with a somatic spike (i.e. no failure) is plotted as a solid line. This is a sigmoidal function with zero spikes at the lowest EPSP amplitudes and zero failures at the highest EPSP amplitudes.

### Absence of spike failures in the medial nucleus of the trapezoid body

**Fig 9:**
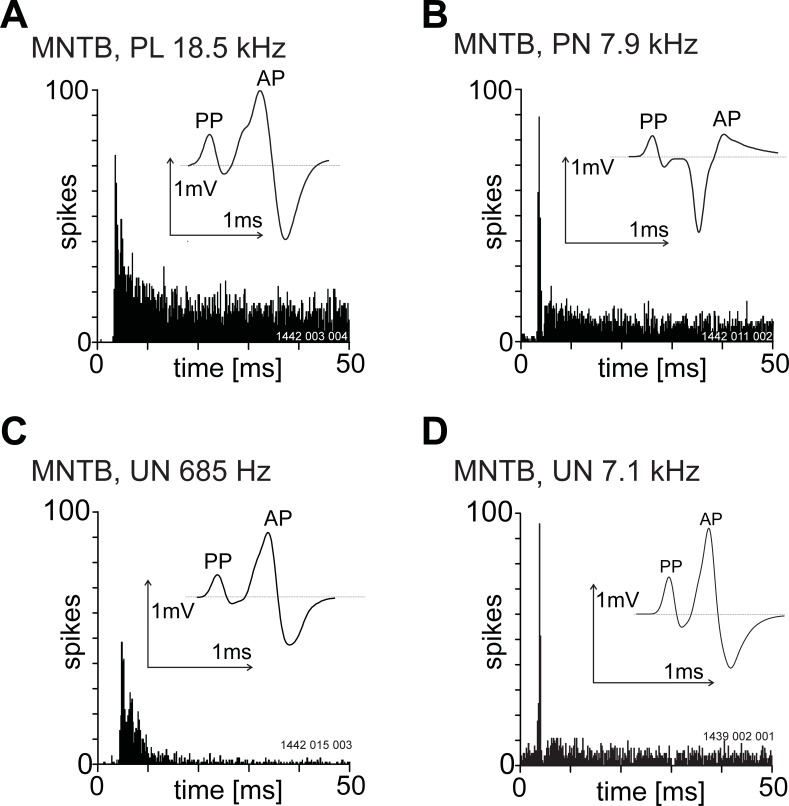
The variety of post-stimulus time histogram shapes in response to 20 dB supra-threshold BF tone bursts from single units recorded from the presumed medial nucleus of the trapezoid body. The unit best-frequency is indicated above each histogram. All histograms were constructed from spike times recorded from the AP-component. Bin-width was 0.2 ms. The stimulus onset was at zero milliseconds and stimulus duration was 50 ms. Inset in each PSTH is the averaged spike waveform shape. Note that in D the pre-potential is larger than the post-synaptic spike. In panel B the AP-component is negative going. UN, unusual.

**Fig 10:**
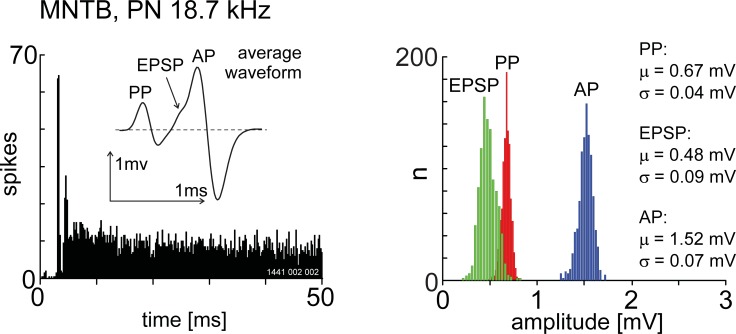
The amplitude distributions for the PP, EPSP and AP components for a unit presumed to be located in the medial nucleus of the trapezoid body. The unit BF was 18.7 kHz and was classified as primary-like with a notch based on the shape of the post-stimulus time histogram (A). The stimulus onset was at zero milliseconds and stimulus duration was 50 ms. Note that the PP is, on average, larger than the EPSP-component.

As part of a study aimed at recording from nuclei in the superior olivary complex we sometimes came across units characterised by an obvious PP which we hypothesize were located within the MNTB. For comparison we present the recordings from 20 of these units, in the same species, under the same anaesthetic regime and using the same type of microelectrode. The variety of PSTH patterns and spike-waveform shapes are shown in Fig **9**. In all cases a PP was followed by an EPSP and AP-component. We did not observe any spike failures in these recordings. Interestingly we did observe different spike waveform shapes. For instance, a PP followed by a negative going spike (Fig **9B**) was never observed in our recordings from the VCN. The PSTH pattern of the majority of the units could be described as either primary-like or primary-like with-a-notch, however, Fig **9C and 9D** shows two units that were unusual in their PSTH shape. Fig **9D**, while displaying a clear notch after the onset peak, could easily be described as an onset responder rather than primary-like with-a-notch. A more conventional primary-like with a notch histogram from the MNTB is shown in Fig 10. The distribution of waveform amplitude was unimodal for all components (see Fig **10B**). Note in this case the mean PP amplitude was larger than the mean EPSP amplitude.

**Fig 11:**
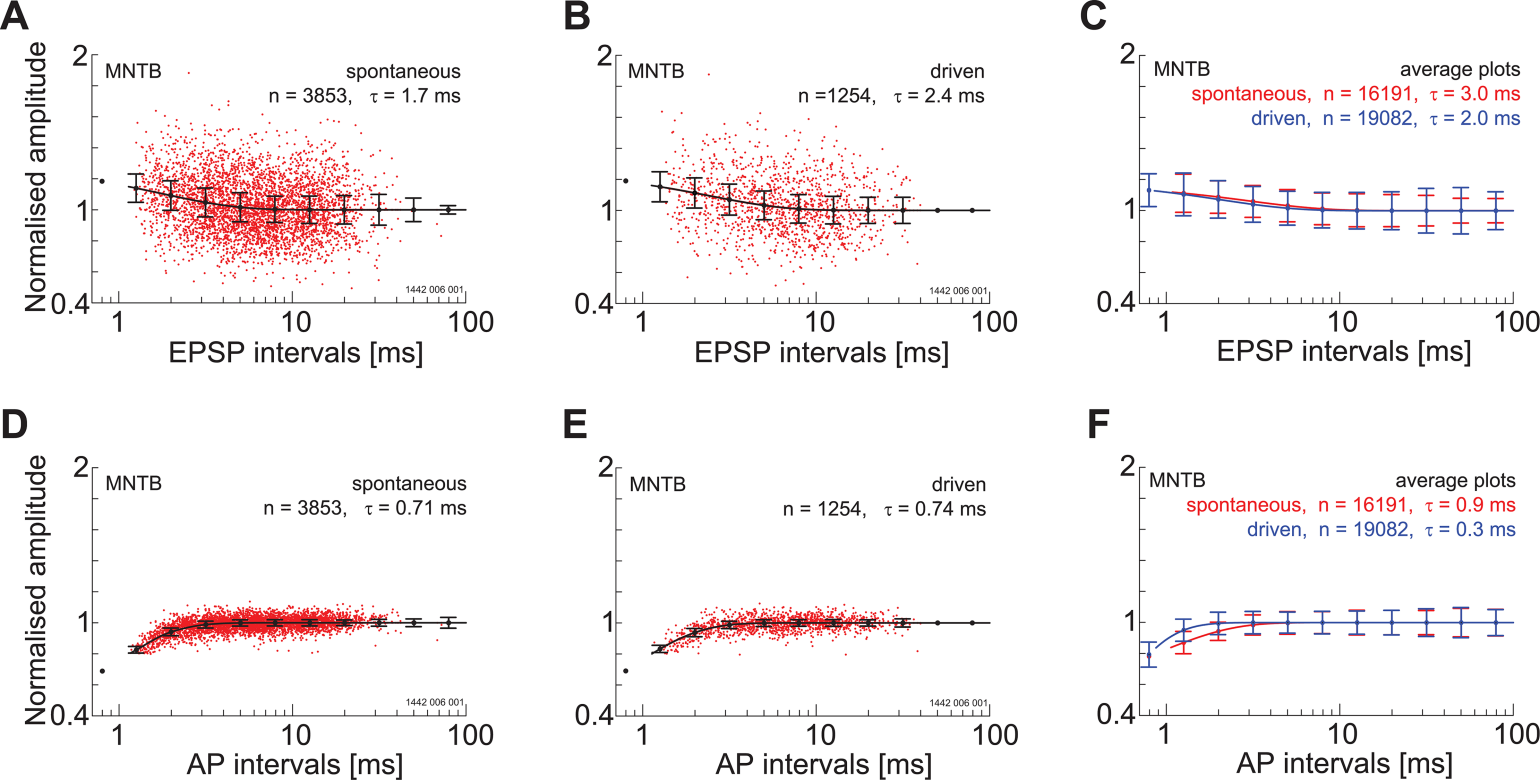
Amplitude of post-synaptic components depends on spike history in the medial nucleus of the trapezoid body. A. Relationship between EPSP-component amplitude and inter-event interval for spontaneous activity for a single unit (PN; BF = 28.4 kHz). Note the facilitation at short intervals. B. A similar response is found for driven activity. C) The response of the population of single units. D-E) Relationship between the AP amplitude and inter-event interval. For both spontaneous activity and driven activity there is a slight reduction in AP amplitude at the shortest inter-event intervals. F. Population response. For all figures the lengths of the vertical lines represent one standard deviation.

In close agreement with the VCN data the amplitude of the EPSP-component shows a facilitation at the shortest inter-event intervals for both driven and spontaneous discharge (Fig **11A and 11B**) **for a single unit** and for a population of units (Fig 11C)). Again in agreement with the VCN data, the AP component amplitude was reduced at the shortest inter-event intervals for both driven and spontaneous activity (Fig 11D and 11E) and for a population of units (Fig 11F).

A comparison of the spike waveform shapes recorded in the VCN and MNTB is provided in **Table 1**. Using a one-way ANOVA (IBM SPSS Statistics 25.0.0) to compare the four classes of unit with a prepotential (PLpp, PNpp, UNpp and MNTB) there was a significant main effect of PP-EPSP transition time (F(3,200) = 27.134; p < 0.05). Using the Games-Howell Post-Hoc test revealed that PP-EPSP interval was significantly smaller for MNTB units than for the other unit types (P < 0.05). There were also significant main effects for PP amplitude (F(3,200) = 88.613; p < 0.05) and EPSP-AP interval (F(3,200) = 27.304; P < 0.05). The Games-Howell Post-Hoc tests revealed significantly larger PP’s and smaller EPSP-AP intervals for MNTB units in comparison with the three other unit classes (P < 0.05). There was no significant main effect for EPSP amplitude (F(3,200) = 2.234; P > 0.05), however, there was a significant main effect for AP amplitude (F(3,200) = 4.463, P < 0.05) with the Games-Howell post-hoc test showing a significant difference in the AP amplitudes for MNTB and PNpp units (P < 0.05).

**Table 1:** A comparison of extracellular waveform statistics.

Unit type		Property				
	Spont. Rate [sp/s]	PP amplitude [mV]	EPSP amplitude [mV]	AP amplitude [mV]	PP-EPSP interval [μs]	EPSP-AP interval [μs]
**MNTB**						
pp [20]	36±38	0.45±0.15	0.64±0.37	1.19±0.55	314±26	127±37
**VCN**						
PL pp [71]	46±39	0.10±0.08	0.61±0.31	1.41±0.58	408±36	200±48
PL [29]	64±43	na	0.69±0.30	1.72±1.03	na	139±37
PN pp [40]	46±46	0.09±0.06	0.50±0.24	1.77±0.83	413±35	228±37
PN [22]	33±38	na	0.79±0.35	2.73±1.72	na	104±60
UN pp [73]	38±35	0.13±0.09	0.65±0.30	1.40±0.64	396 ±57	209±38

Note that both the PP-EPSP interval and the interval between the EPSP and AP is significantly less for the units recorded from the MNTB. The amplitude of the MNTB PP is significantly greater than the PP amplitudes found for the VCN units. Spontaneous rate and component intervals are given to the nearest integer. All peak amplitudes are given to 2 dp. Numbers in square brackets are total number of units. na = not applicable

### EPSP and AP components are observable in non-PP units in the VCN

**Fig 12:**
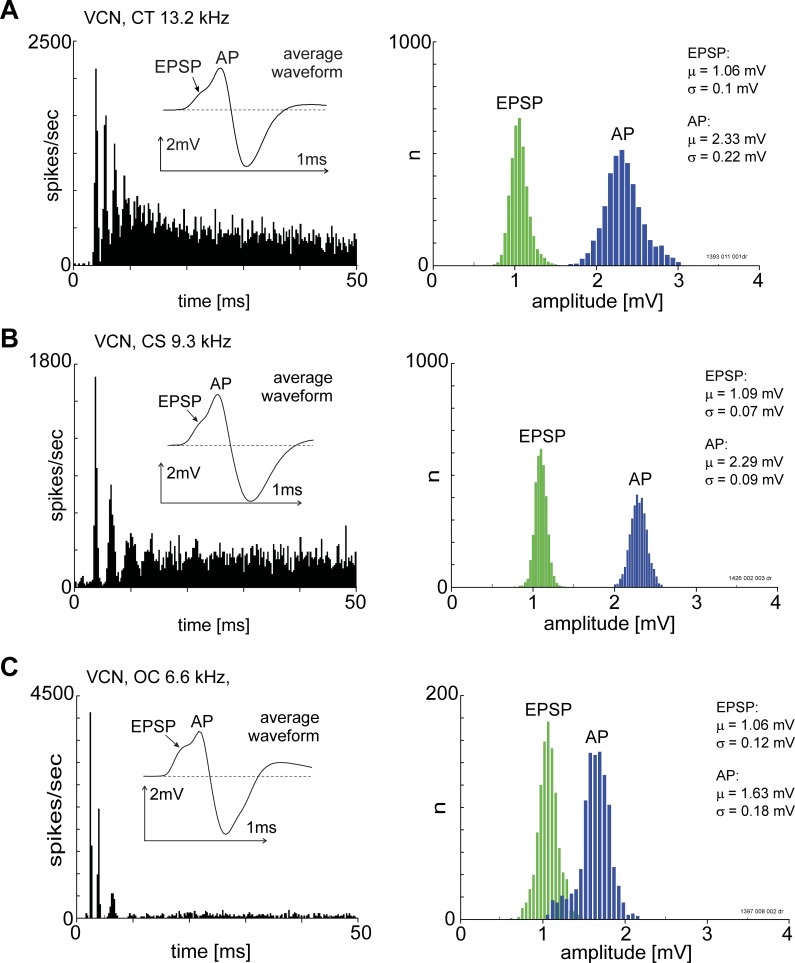
PSTHs, averaged spike waveforms and spike amplitude distributions for three units without a pre-potential or primary-like PSTH shape. The left-hand column shows spike waveforms (inset) with a discernible EPSP and AP components. The units were classified as transient chopper (top row), sustained chopper (middle row) and onset-chopper (bottom row). Unit BF is indicated at the top of each plot in the left column. The unit best-frequency is indicated above each histogram. All histograms were constructed from spike times recorded from the AP-component. Bin-width was 0.2 ms. The stimulus onset was at zero milliseconds and stimulus duration was 50 ms.

Fig **12** shows data from three example units recorded in the VCN. These units are non-PP types, recorded from presumed multipolar cells. These neurons do not receive giant synapses. Each of these units has large somatic action potentials and EPSPs.

## Discussion

### Summary of results

All units with a PP recorded from the VCN of the anaesthetised guinea pig showed a failure of the AP-component in the spike waveform. The mean failure rate when estimated from spontaneous discharge was 59%. This contrasts with the MNTB in the same species, under the same anaesthetic regime and similar recording conditions, where no failures were observed. Failures were also common in the VCN during driven activity. Both the shape of the receptive field and the temporal adaptation pattern were altered. In the VCN, we also recorded data from units which had primary-like PSTHs but no PP. These units did not show spike failures, but could nevertheless be classified as primary-like, primary-like-with-a-notch, or unusual, and accounted for ~30% of our total unit sample. Crucial to our interpretation of these results is an understanding of the origin of the complex waveform shape.

### The origin of the complex waveform shape

We (and others) have argued strongly that the 2 and 3 component waveform shapes are recorded from the same neuron. As discussed in the methods section it is ultimately impossible to prove that the waveforms came from the same cell using our method of recording but it should be noted that failures have been observed when recording intra- and extra-cellular potentials simultaneously from the same cell [22]. Ultimately, even if we were recording from two neurons this would still leave us having to explain why there are two types of neuron with a pre-potential; one that resembles a primary afferent fibre in its responses while one looks distinctly non-primary-like. It should also be noted that only units with a pre-potential showed spike failures. We are therefore confident that the only anatomical cell types that shows spike failures are bushy cells in receipt of end-bulb, or modified end-bulbs, of Held synapses.

### Mechanisms of spike failure

The failure of the AP component meant that traditional PSTH patterns and receptive fields were substantially altered. The failure of the AP component of the complex waveform recorded extra-cellularly from cells with a pre-potential observed in the AVCN of the cat was first described by Pfeiffer [19]. This observation was followed up by a large study of recordings from the AVCN, also in the cat, by Bourk [18] who described two types of pre-potential, PP1 and PP2. For PP1 units the pre-potential was clearly visible in the raw (single-trial unaveraged) spike waveforms whereas the spike waveform needed to be averaged across multiple spikes to observe a pre-potential in PP2 units. The overwhelming majority of single units with a pre-potential could be classified as primary-like or primary-like with a notch. There were, however, several units that were either identified as On-P (onset-primarylike) or Pri-LR (low steady-state discharge rate). The On-P or Pri-LR could be similar to the unusual types that we describe here.

The majority of the recordings by Bourk [18] consisted of a small positive component—the PP—followed by a negative going post-synaptic event, less than 1 mV in amplitude. Bourk [18] also reported a few examples of what he termed “giant spikes” which were positive going and greater than 1 mV in amplitude. In one example Bourk [18] noted that the AP component disappeared as the electrode approached the cell with the implication being that this was presumably due to damage to the cell body. While it is impossible to refute totally this idea several observations suggest this is unlikely: (a) the units reported in this paper were remarkably stable for several hours; no increase in discharge rate was noted as the amplitude of the action potential grew (b) presumed damage to neurons was observed when advancing the electrode as an increase in spike discharge rate, both driven and spontaneous rates (c) when a unit was lost both the three component waveforms and the two component waveforms (spike failures) disappeared simultaneously.

Subsequent reports of units with a pre-potential but non-primary-like PSTHs were reported in the cat [31] and in the guinea pig [30, 32]. The results from the guinea pig also showed that the receptive field of these units was very unusual with large amounts of suppression, both centre-band and side-band. The rate-level functions of these units were often non-monotonic. They reported that these responses occurred in about 27% of their population. Subsequently these histogram and receptive field changes were ascribed to spike failures by Kopp-Scheinpflug et al [16] in the AVCN of the gerbil. In this study, presumed spherical bushy cells (corresponding to primary-like units) were found to integrate neural inhibition with excitatory inputs from ANFs. This inhibition was tuned, resulting in non-monotonic rate level functions and a transformation from a type-I receptive field in the ANF inputs to a more complex receptive field in the AVCN output.

It has now become clear that the factors determining the reliability of the endbulb synapse in the VCN are the fluctuations in EPSP amplitude, and that during acoustic stimulation hyperpolarizing inhibition interacts with depolarising endbulb input to reduce spike firing. This inhibition increases in strength with increasing sound intensity. It decreases the rising slope of the EPSP, and the EPSP-AP transition time. Some studies [24, 33] suggest that the inhibitory and excitatory inputs are co-tuned, while others [34] have found less overlap. It is likely that both co-tuning (“centre-band inhibition”) and non-co-tuning (“side-band inhibition”) could occur in the same species [16, 30]. The short delay between the onset of inhibition (approximately 1 ms) compared to the onset of excitation suggests just one extra synapse in the inhibitory circuit. The obvious place to examine for the origin of the inhibition is the cochlear nucleus. One possible source of side-band inhibition is the D-multipolar neurons. These neurons are characterised by broadly-tuned receptive fields and have higher thresholds than narrowly-tuned cells in the same region [35]. A source of centre-band inhibition visible extra-cellularly could be the tuberculo-ventral cells in the dorsal cochlear nucleus [36], however, this does not preclude sources from outside the cochlear nucleus, e.g., the superior olivary complex.

### Recordings from the trapezoid body

If the failure of the AP component represents the failure of spike initiation, then it should follow that the responses in the output fibres from PL and PN units should also be non-primary-like. The trapezoid body (*aka* the ventral acoustic stria) is the major output pathway for neurons in the VCN, including those classified as PL or PN and it is therefore apposite to examine the reports of recordings from this fibre tract [37–40]. While all studies described the responses of fibres in the trapezoid body as being primary-like, some of these reports showed that responses recorded from globular bushy cells were characterised by PSTH patterns that were onset-like in shape. These onset-like units (Fig 1C, 1D and 1E in Smith et al., [38]; Fig 7C and 7E in Spirou et al., [39]) are similar in shape to the PSTHs shown in Figs 4 and 9 here. As the onset-like PSTH shapes were produced by spike failure in this paper it is possible that the onset-like discharge patterns were also characterised by spike failures. Alternatively the onset-like PSTHs reported by Smith et al. [38] from recordings in the trapezoid body were not due to spike failures *per se* in globular bushy cells but rather to intrinsic cell properties. For example, Spirou et al. [41] and Banks and Sachs [42] have demonstrated that it is possible for computational models of globular bushy cells to exhibit onset-like PSTH shapes.

### Implications for signal processing

The existence of large synapses in the auditory pathway has hitherto been assumed to indicate the importance of preserving timing information present in their ANF input [19, 30, 43]. Recordings from primary-like units in the AVCN (most-likely spherical bushy cells) demonstrate their ability to represent the spectrum of vowel sounds in their temporal discharge patterns [9, 10]. It has also been shown that similar cells can encode the pitch of complex sounds in their inter-spike intervals [e.g., 8]. Compared to ANFs, VCN primary-likes have strong, or even enhanced, synchronization to single tones and complex sounds [30, 43–45].

In the gerbil it has been shown that PP primary-like units with spike failures have enhanced phase-locking to tones and complex sounds [33, 46–48]. This enhancement has also been shown in a computational model of the response patterns seen in the gerbil. Of course, if the failure of spikes is an adaptation for improved temporal performance then it must be asked why we do not observe this enhancement in all primary-like units. Are there different populations of primary-like units? This has been hinted at for some time by the observation by Osen [49] of two different sizes of spherical bushy cell in the AVCN of the cat. The difference between the large and small spherical bushy cells was never clearly delimited and it has subsequently been shown in the guinea pig [50] and the gerbil [51] that while there are differences in size, the distribution is not bimodal. Rather there is a continuum of cell sizes amongst the spherical bushy cell class. Spherical bushy cells project to the MSO, LSO and other areas in the superior olivary complex. It is possible that different types of spherical bushy cell project to the different SOC nuclei.

It is clear from the results reported here and from the data from several laboratories that it is still uncertain as to precisely the information leaving the antero-ventral cochlear nucleus. Until this issue is resolved, models of VCN processing based exclusively on auditory-nerve responses are potentially misleading.

## Abbreviations

ANF: auditory nerve fibre
AP: action potential
AVCN: antero-ventral cochlear nucleus
BF: best frequency
CAP: compound action potential
DCN: Dorsal cochlear nucleus
EPSP: excitatory post-synaptic potential
MNTB: medial nucleus of the trapezoid body
MSO: medial superior olive
LSO: lateral superior olive
PP: pre-potential
PL: primary-like
PN: primary-like with a notch
SOC: superior olivary complex
UN: unusual
VCN: ventral cochlear nucleus
